# International Comparative Study of Statistics Learning Trajectories Based on PISA Data on Cognitive Diagnostic Models

**DOI:** 10.3389/fpsyg.2021.657858

**Published:** 2021-07-20

**Authors:** Bing Jia, Zhemin Zhu, Hang Gao

**Affiliations:** ^1^School of Mathematics and Statistics, Northeast Normal University, Changchun, China; ^2^School of Education Science, Beihua University, Jilin, China

**Keywords:** PISA, learning trajectories, international comparison, statistics education, cognitive diagnosis

## Abstract

The cognitive diagnosis model is an emerging evaluation theory. The mastery of fine-grained knowledge points of students can be obtained *via* the cognitive diagnostic model (CDM), which can subsequently describe the learning trajectory. The latter is a description of the learning progress of students in a specific area, through which teaching and learning can be linked. This research is based on nine statistical items in the Program for International Student Assessment (PISA) 2012 and an analysis of the response data of 30,092 students from 14 countries from four attributes based on CDM. Then, it obtains the learning trajectory of students in statistical knowledge. The study found that Bulgaria, Costa Rica, Peru, Mexico, and Serbia have the same learning trajectories. The learning trajectories of almost 14 countries are as follows: (1) uncertainty, (2) data handling, (3) statistical chart, and (4) average.

## Introduction

Over the past century, the need for statistical knowledge has grown; therefore, an increasing number of statistics knowledge has been presented to K-12 classrooms (Schaeffer and Jacobbe, 2014). For example, in the United States, the Common Core State Standards for Mathematics (CCSSM, National Governors Association Center for Best Practices and Council of Chief State School Officers, 2010) call for students to create and interpret data displays beginning in grade 1. These changes put forward demands on statistical education. Accordingly, statistics education is developing as a new and emerging discipline (Garfield and Ben-Zvi, 2008). Statistics education research has emphasized the need for reform in the teaching method, educational concept, and teaching tools to improve the teaching and learning effect of statistics (Tishkovskaya and Lancaster, 2012). However, research on the teaching and learning of statistics remains disconnected, fragmented, and difficult to access (Zieffler et al., 2008). A gap remains between the research and practice of teaching statistics (Tishkovskaya and Lancaster, 2012). Learning trajectories are a link between teaching and learning. The researcher can obtain information about how to teach through learning trajectories.

Learning trajectories contain a description of the dynamic process of learning. The first aspect of learning trajectories is a specified learning model. It reflects the natural developmental progressions identified in theoretically and empirically grounded models of thinking, learning, and development of students (Carpenter and Moser, 1984; Griffin and Case, 1997). The second aspect of learning trajectories is an instructional sequence. It is used to identify tasks as effective in determining the content of the course. In general, consistent learning with this learning trajectory is believed to be more effective for students than learning that does not follow these learning trajectories (Clements and Sarama, 2004). The learning trajectory can be obtained from the continuous observation of the learning process of the individual, or it can be obtained *via* quantitative research on a large number of tests for students. Learning trajectories through quantitative research can be obtained by using two types of cognitive diagnostic model (CDM): models based on item response theory, such as rule-space model (RSM) and attribute hierarchical model (AHM), and models that are not based on item response theory.

Cognitive diagnostic model can provide fine-grained feedback by pinpointing the presence or absence of multiple fine-grained skills or attributes (Templin and Bradshaw, 2013; Zhang and Wang, 2020). This research follows the existing CDM method (Wu et al., 2020) to seek the learning trajectory of statistical knowledge. This research aims to employ a CDM as an analytic tool to analyze the Program for International Student Assessment (PISA) dataset consisting of 14 countries, including the United Arab Emirates, Argentina, Bulgaria, Chile, Colombia, Costa Rica, Jordan, Kazakhstan, Mexico, Peru, Romania, Serbia, Tunisia, and Uruguay on the statistics items in the PISA test contents. First, the PISA test data and cognitive diagnosis models are introduced. The second part introduces the process of analyzing data. The third part provides the trajectory of statistical content learning in different countries. Finally, the learning trajectory is analyzed.

## Program for International Student Assessment

Program for International Student Assessment, as one of the most influential educational assessment programs in the world, assesses science, reading, and mathematics literacy of students. The PISA takes place every 3 years, with the most recent one held in 2018. In the education system, approximately 80 countries and regions with 600,000 students participated in PISA 2018. However, PISA 2018 checks reading literacy, and no public math items were included. The most recent math test item was opened in 2012. PISA 2012 assessed reading, mathematics, and science literacy (with a focus on mathematics) of approximately 510,000 students as a whole representing about 28 million 15-year-olds globally in 65 countries and economies. Each country or economy only uses part of the test items in the item bank. Therefore, not all students from all countries and economies use the same items. As one of the most influential educational assessment programs in the world, PISA has had a large impact on educational practice and reform in many countries by increasing the scopes of tests and strengthening the interpretation of results; consequently, it influences the decision-making processes for the improvement of national education policies (Breakspear, 2012; OECD, 2013; Wu et al., 2020).

The analysis of the characteristics of education in various countries through the PISA project is indispensable in the promotion of the reform and development of mathematics education (Wu et al., 2020). Research based on PISA mathematics scores can be divided into three categories. The first category is the study of factors affecting the mathematical literacy of students. Examples are gender differences in PISA performance (Liu et al., 2008), the impact of information and communication technology (ICT) (Zhang and Liu, 2016), and the learning trajectory research (Wu et al., 2020). The second category is the study of mathematics learning psychology. Examples are math anxiety (Foley et al., 2017; Luttenberger et al., 2018), math self-concept and math self-efficacy (Lee, 2009), and motivation (Oden, 2020). The third category is the study of the test items themselves. Examples are item development (Dasaprawira, 2019) and research on other mathematics items based on PISA mathematics literacy (Öztürka and Masalb, 2020). These studies are based on classical measurement theory and item response theory because the Organization for Economic Cooperation and Development (OECD) uses IRT to overcome the limitations of scoring methods on the bases of correct number or correct percentage (OECD, 2015). Some studies also use CDM (Leighton et al., 2010; Wu et al., 2020).

It is necessary to study the statistical items separately. In the above studies, statistical items are considered as a whole under the mathematics items, and statistical projects are rarely studied separately. Although statistics and mathematics have many connections, statistics is a separate discipline and is not a branch of mathematics. Considering that statistics and mathematics are different in the crucial role of context, issues of measurement, the importance of data collection, and conclusions are either definitive or not (Rossman et al., 2006). For example, educational research shows that students (and others) experience profound difficulties with reasoning under uncertainty (Garfield and Ahlgren, 1988; Shaughnessy, 1992; Garfield, 1995). Therefore, from an empirical point of view, the experience and reaction of students who are learning statistics are different from those learning mathematics.

## Cognitive Diagnosis Model

The construction of the CDM is based on two elements. One element is an item and attribute association matrix called *Q*-matrix (Tatsuoka, 1983). Simply put, the *Q*-matrix is to calibrate which attributes (e.g., skills and knowledge) are examined for each topic. The *Q*-matrix is typically developed by domain experts and assumed to be correct in the following CDM analyses (Ma and de la Torre, 2020). The second element is a list of models used to identify the potential cognitive characteristics or skill mastery patterns of students.

### Attributes in CDM

The most significant difference between IRT and CDM is that IRT assumes a continuous latent factor called ability, whereas CDM is a structured latent class model which assumes a discrete latent variable called attributes. Attributes are a set of fine-grained skills typically designed to provide diagnostic information about the strengths and weaknesses of students. The major goal of CDM analyses is to infer the attribute profiles of students from their item responses (Ma and de la Torre, 2020). After mastering the attributes and characteristics of students, teachers can conduct targeted teaching. If students understand their own attributes, they can compensate for their learning weaknesses. Furthermore, researchers can search for general rules from the attribute profiles of a large number of students to explore possible learning trajectories (e.g., Wu et al., 2020).

Cognitive diagnostic models infer the mastery of the attributes of the examinees through the *Q*-matrix and the responses of the examinees. The *Q*-matrix is a matrix that describes what attributes are examined by each item in an examination. For example, *Q*-matrix:

$$Q=\left(\begin{array}{c} 1 1 1 0\\ 0 1 0 1 \end{array}\right)$$

indicates two items examining four attributes. The first question examines the first three attributes, while the second question examines the second and fourth attributes.

### Cognitive Diagnosis Models

Cognitive diagnostic models include three different types of models: the compensatory, non-compensatory, and general models. In the compensatory model, one or some attributes of the examinee can make up for the deficiencies of other attributes. This statement means that, if a question checks multiple attributes, the participant needs to master only one of them. It is generally used more in psychology. For example, anxiety and depression can all cause insomnia. By contrast, the lack of mastery of one attribute of the non-compensatory models cannot be completely compensated by other attributes in terms of question performance, that is, all the attributes must function in conjunction with each other to produce the correct answer (Ravand and Robitzsch, 2015). The general model generalizes the formula, so that the CDM allows two types of relationships in the same test.

The most famous compensatory model is the deterministic input noisy-or-gate (DINO) model (Templin and Henson, 2006). The most famous non-compensatory (conjunctive) model is the deterministic input noisy-and-gate (DINA) model (Junker and Sijtsma, 2001). DINA model is one of, if not the simplest, consequently most restrictive, the interpretable CDMs available for dichotomously scored test items (de la Torre, 2011). It contains two item parameters, namely, guessing and slipping parameters. The Reduced Reparameterized Unified Model (R-RUM) (Hartz et al., 2002) is a latent class conjunctive model because it assumes that the latent ability space of students can be dichotomized into mastery and non-mastery and that students must master all required skills to obtain a correct item (Roussos et al., 2007). The most famous general model is the general DINA (G-DINA) model (de la Torre, 2011). The DINA model and the DINO model can be obtained from the G-DINA model by setting parts of the parameters to zero. In addition, the additive CDM (A-CDM; de la Torre, 2011) assumes that each required attribute uniquely and independently contributes to the success probability. The LLM is the logit link G-DINA model without any interaction terms (Maris, 1999). Log-linear model with latent variables for cognitive diagnosis (LCDM; Henson et al., 2009) is a general version of the RUM.

In mathematics education, non-compensatory and general models are typically used. To illustrate, for a mixed operation of addition and subtraction, students are most likely to perform it correctly after they have mastered addition and subtraction. Therefore, if students do not know one knowledge point (subtraction) in a mixed operation problem, using another knowledge point (addition) to solve a problem is difficult. The same is true in statistics. If a student cannot calculate the average, then he cannot use uncertainty to solve this problem. Therefore, only non-compensatory and general models were considered in this research.

## Materials and Methods

In PISA 2012, students from 14 countries answered the same nine statistical questions. Although other countries have also examined some statistical questions, they are generally less than four questions and do not have the conditions to use CDMs. So, this research gathered a total of 30,092 datasets from 14 countries [e.g., United Arab Emirates (AE), Argentina (AR), Bulgaria (BG), Chile (CL), Colombia (CO), Costa Rica (CR), Jordan (JO), Kazakhstan (KZ), Mexico (MX), Peru (PE), Romania (RO), Serbia (RS), Tunisia (TN), and Uruguay (UY)]. These datasets can be downloaded from the Supplementary Material. These 14 countries share nine projects in PISA 2012: PM942Q01, PM942Q02, PM957Q01, PM957Q02, PM957Q03, PM985Q01, PM985Q02, and PM985Q03.

This study defines attributes based on the content area which is given by PISA and specific statistical attribute indicators as four different statistical knowledge points: uncertainty, average, statistical chart, and data handling. These four attributes are important knowledge points in statistics textbooks for elementary and junior high schools. Table 1 shows the corresponding definitions of attributes.

**TABLE 1:** Dimensions of cognitive attributes.

**Attribute**	**Definition**
Uncertainty	Perception of change, probability and opportunity, representation, evaluation, interpretation of uncertainty-centric data
Average	Calculate the average, understand the meaning of the average, and use the average to describe and explain the actual problem
Statistical chart	Use statistical tables to organize data and read data from statistical tables
Data handling	Compare different data, use numbers to represent the quantity, and get the quantitative relationship from the numbers

These four attributes are taught in elementary education textbooks in nearly all countries. Through discussions with statistical education experts and middle school teachers, the attributes of each topic were determined. Table 2 shows the specific attributes of each topic.

**TABLE 2:** Q-matrix of 9 test items in PISA.

**Items**	**Attributes**			
	**Uncertainty**	**Average**	**Statistical chart**	**Data handling**
PM942Q01	1	1	0	0
PM942Q02	0	1	0	1
PM942Q03	0	1	0	0
PM957Q01	0	1	0	1
PM957Q02	0	1	0	0
PM957Q03	0	1	0	1
PM985Q01	1	0	1	1
PM985Q02	0	0	1	1
PM985Q03	0	0	1	0

A large number of cognitive diagnostic practices have shown that choosing an appropriate cognitive diagnostic model is an important prerequisite for an accurate diagnosis or classification of subjects (Tatsuoka, 1984). Existing studies have shown that CDM is suitable for PISA 2012 (e.g., Wu et al., 2020); thus, Absolute Fit Index, which is used to judge whether CDMs fit the data, such as chi-square, is not used in this research. Relative fit indexes, Akaike’s information criterion (AIC), and Bayesian information criterion (BIC) are used as reference standards. Relative fit indexes are used to determine which model in the model set fits the data better. Owing to a collection of competing models for the data, the AIC estimates the quality of each model relative to those of other models. The BIC is a criterion for model selection among a finite set of models, and the model with the lowest BIC value is preferred (Zhu et al., 2019). In the above, six kinds of different CDMs (DINA, ACDM, GDINA, LLM, LCDM, and RRUM) were evaluated. The model with the least BIC has a better fit than the other models.

This research used statistical language *R* and R packages GDINA in software RStudio (version 1.2.5033). The package and reference manual can be obtained from https://cran.r-project.org/web/packages/GDINA/. Through the GDINA packages, the attribute patterns of 30,092 students from 14 countries and AIC and BIC of each model can be estimated.

These attribute patterns were used to determine the statistics learning trajectory. The procedure has two steps. First is creating a hierarchy of knowledge states based on their logical dependencies. The attribute pattern is a vector of 0 or 1. Among them, 1 means that the student has mastered this attribute, and 0 means that the student has not yet mastered the corresponding attribute. It tells the examiners or teachers what attributes the students have mastered as well as the defects in which attributes. For example, (1,1,1,1) indicates that the student has mastered all four attributes, and (0,0,0,1) indicates that the student has mastered only the data handling attribute. If the attribute(s) mastered in one attribute pattern is a subset of the attributes mastered in another attribute pattern, then they must have a logical relationship. For example, the attribute patterns (1,0,0,0) and (0,0,0,1) are the prerequisites of the attribute pattern (1,0,0,1). That is, if the student wants to master the first and fourth attributes simultaneously, he/she must first master the first attribute or the fourth attribute. Therefore, a containment relationship exists between the two knowledge states, that is, the hierarchy of knowledge states is (1,0,0,0) → (1,0,0,1) or (0,0,0,1) → (1,0,0,1). Second, the learning trajectory is the path of more frequent states across this hierarchy. Students in every country have a variety of mastery attribute patterns. The frequency of the mastery attribute pattern is different. Empirically, the patterns with more frequency of teaching are most consistent with the statistical education of the country; the patterns with less frequency may be caused by random errors. Therefore, this study takes the more frequent attribute mastery patterns in each country as the main research object. The hierarchical structure of the knowledge state inferred based on these attribute patterns is the path of statistical learning of students, which means the learning trajectory. This method to get a learning trajectory is called the logistic CDM method (LCM).

## Results and Research Analysis

The research objects are the responses of a total of 30,092 students from 14 countries on nine items. Table 3 lists the descriptive statistics on the number of correct answers for all students.

**TABLE 3:** The Number of correct answers in 9 items for all students.

**Total**	**Frequency**	**Percent**	**Valid percent**	**Cumulative percent**
0	1,552	5.2%	5.2%	5.2%
1	5,610	18.6%	18.6%	23.8%
2	7,316	24.3%	24.3%	48.1%
3	6,223	20.7%	20.7%	68.8%
4	4,284	14.2%	14.2%	83.0%
5	2,726	9.1%	9.1%	92.1%
6	1,468	4.9%	4.9%	97.0%
7	644	2.1%	2.1%	99.1%
8	212	0.7%	0.7%	99.8%
9	57	0.2%	0.2%	100.0%

Table 3 indicates that nearly half of the students only obtained two correct items, and 83% correctly answered less than half of the questions. Only 0.2% of the students correctly answered all the questions. Thus, the scores of students from these 14 countries are not ideal on statistical items. Table 4 shows the result of model fit.

**TABLE 4:** Parameter statistics comparison of different models.

**Models**	**Deviation**	**AIC**	**BIC**
DINO	223233.2	223299.2	223569.3
DINA	223317.0	223383.0	223653.2
GDINA	222641.9	222739.9	223141.0
ACDM	222690.4	222770.4	223097.9
LLM	222687.4	222767.4	223094.8
RRUM	222704.9	222784.9	223112.3
LCDM	222642.0	222740.0	223141.2

The results in Table 4 showed that LLM has a better fit than the other models because the value for BIC of the LLM is the smallest. Therefore, LLM was preliminarily selected. According to the results of the above model selection, LLM is used as the cognitive diagnosis model of this study to estimate the attribute pattern of the student.

### Attribute Mastery Probability

**FIGURE 1:**
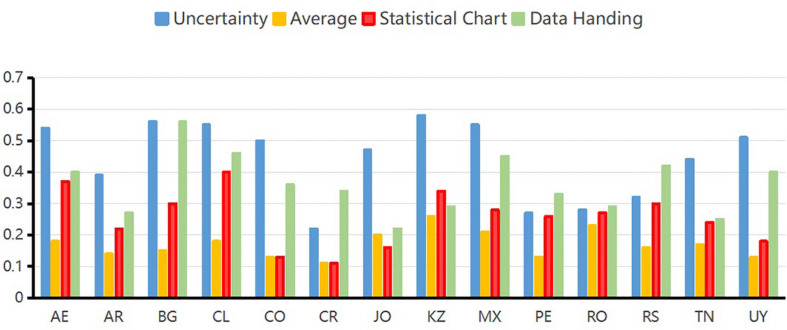
Average of expected a posteriori of each attribute for all students in each country.

In the GDINA package, the probability that the student masters the knowledge states can be obtained from the expected a posteriori (EAP) by the function “personparm.” That is, the probability of a student mastering a certain attribute means the knowledge states of students. Table 5 and Figure 1 show the average values of the EAP of knowledge status of students of four attributes in 14 countries.

**TABLE 5:** Average value of the EAP in 14 countries.

**Country**	**Uncertainty**	**Average**	**Statistical chart**	**Data handling**
United Arab Emirates	0.54	0.18	0.37	0.40
Argentina	0.39	0.14	0.22	0.27
Bulgaria	0.56	0.15	0.30	0.56
Chile	0.55	0.18	0.40	0.46
Colombia	0.50	0.13	0.13	0.36
Costa Rica	0.22	0.11	0.11	0.34
Jordan	0.47	0.20	0.16	0.22
Kazakhstan	0.58	0.26	0.34	0.29
Mexico	0.55	0.21	0.28	0.45
Peru	0.27	0.13	0.26	0.33
Romania	0.28	0.23	0.27	0.29
Serbia	0.32	0.16	0.30	0.42
Tunisia	0.44	0.17	0.24	0.25
Uruguay	0.51	0.13	0.18	0.40

As can be seen from the EAP map of content attributes in Figure 1, the United Arab Emirates, Bulgaria, Chile, Colombia, Kazakhstan, Mexico, and Uruguay have better performances than other countries in uncertainty attribute. The probabilities of mastering uncertainty attribute in these seven countries are higher than 0.5. The probability of mastering average attribute for students of Kazakhstan, Mexico, and Romania are higher than students of other countries. However, the probability of mastering average attribute is not good enough in all these 14 countries. Chile performed the best in mastering statistical chart attribute. Bulgaria performed the best in mastering data handling attribute. Overall, Chile has the best overall performance in the four attributes, followed by Bulgaria. Costa Rica had a low level of mastery average performance on the four attributes. Among the four attributes, the probability of mastering average attribute is the worst performance in 14 countries. Introductory statistics textbooks tend to use the word “average” to describe the process of determining the mean of a dataset (Kaplan et al., 2010). However, the term “average” is occasionally used for any measure of center and at times used for the mean (Triola, 2006). Thus, confusion of concepts in practical problems may be the reason why students are not very accurate on the average items in the actual situation.

### Attribute Pattern

In the GDINA package, the attribute patterns can be obtained *via* three estimation methods: expected a posteriori (EAP) estimation, maximum a posteriori (MAP) estimation, and maximum likelihood estimation (MLE). This research used EAP to estimate the attribute patterns of 30,092 students in 14 countries. EAP is expected to be simple, efficient, and stable, and it is a better choice in the capacity parameter estimation method (Chen and Choi, 2009; Wu et al., 2020). Moreover, it is the default method of the GDINA package. Attribute pattern can be obtained by EAP. This part first classifies the attribute pattern of each student, calculates the proportion of corresponding attribute patterns, and counts the top five attribute patterns in 14 countries as shown in Table 6. Obviously, the attribute patterns with a high proportion correspond to the main knowledge mastery situations of students in each country.

**TABLE 6:** The five most frequency patterns in each country.

**Country**	**1st**		**2nd**		**3rd**		**4th**		**5th**	
	**Pattern**	**Rate**	**Pattern**	**Rate**	**Pattern**	**Rate**	**Pattern**	**Rate**	**Pattern**	**Rate**
United Arab Emirates	(0,0,0,0)	0.363	(1,0,0,0)	0.112	(1,0,0,1)	0.112	(1,1,1,1)	0.100	(0,0,1,0)	0.007
Argentina	(0,0,0,0)	0.494	(1,0,0,0)	0.240	(1,0,0,1)	0.060	(1,0,1,1)	0.059	(1,1,1,1)	0.053
Bulgaria	(0,0,0,0)	0.234	(1,0,0,1)	0.212	(0,0,0,1)	0.166	(1,1,1,1)	0.130	(1,0,0,0)	0.103
Chile	(0,0,0,0)	0.389	(1,0,1,1)	0.222	(1,0,0,0)	0.155	(1,1,1,1)	0.113	(1,0,0,1)	0.065
Colombia	(0,0,0,0)	0.479	(1,0,0,1)	0.178	(1,0,0,0)	0.138	(1,1,1,1)	0.051	(0,0,0,1)	0.037
Costa Rica	(0,0,0,0)	0.645	(1,0,0,1)	0.151	(0,0,0,1)	0.061	(0,1,1,0)	0.034	(1,0,0,0)	0.025
Jordan	(0,0,0,0)	0.511	(1,0,0,0)	0.196	(1,1,0,1)	0.051	(0,0,1,0)	0.051	(1,1,1,1)	0.051
Kazakhstan	(0,0,0,0)	0.332	(1,0,0,0)	0.248	(1,1,1,1)	0.118	(1,0,1,1)	0.070	(1,0,1,0)	0.033
Mexico	(0,0,0,0)	0.415	(1,0,0,1)	0.159	(1,0,0,0)	0.129	(1,1,1,1)	0.114	(1,0,1,1)	0.085
Peru	(0,0,0,0)	0.603	(1,0,0,1)	0.112	(1,0,1,0)	0.056	(1,1,1,1)	0.045	(0,0,0,1)	0.040
Romania	(0,0,0,0)	0.510	(0,1,1,1)	0.143	(1,0,0,1)	0.132	(0,0,1,0)	0.080	(1,0,0,0)	0.048
Serbia	(0,0,0,0)	0.422	(1,0,1,1)	0.112	(1,0,0,1)	0.108	(1,0,0,0)	0.075	(0,0,0,1)	0.073
Tunisia	(0,0,0,0)	0.490	(1,0,0,0)	0.202	(1,1,1,1)	0.090	(1,0,0,1)	0.059	(0,0,0,1)	0.049
Uruguay	(0,0,0,0)	0.459	(1,0,0,0)	0.189	(1,0,0,1)	0.122	(1,0,1,1)	0.097	(1,1,1,1)	0.049

Table 6 shows that the first attribute pattern is (0,0,0,0), indicating that most students have not yet mastered the attribute patterns of all content attributes. It is followed by attribute patterns (1,0,0,0) and (1,0,0,1), which are relatively close. The hierarchy of knowledge states can be based on their logical dependencies. Based on the logical relationship between high-frequency attribute patterns, the learning trajectory can be confirmed.

### Learning Trajectories

**FIGURE 2:**
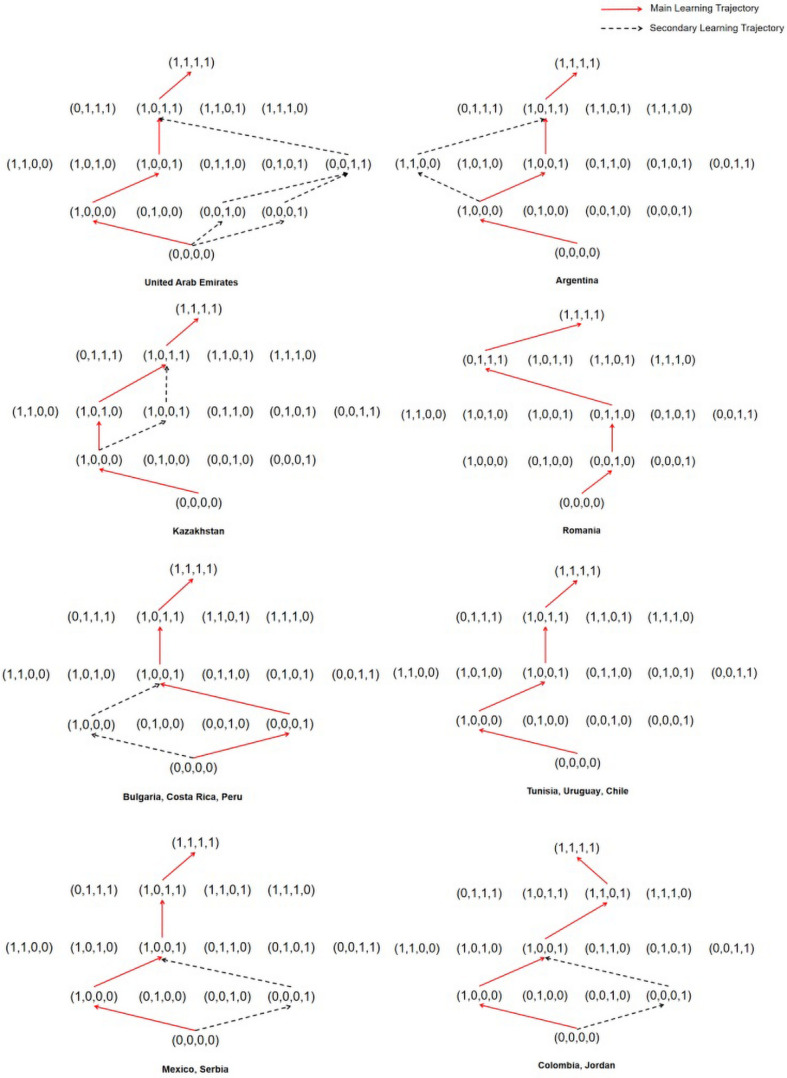
Figures of learning trajectories.

Learning trajectories are a description of qualitative change in the level of sophistication of a student for a key concept, process, strategy, practice, or habit of mind (Deane et al., 2012). In the process of establishing a learning trajectory, students are generally assumed to learn from relatively simple and then learn more difficult knowledge because mastering lower-level attributes should be easy and mastering higher-level attributes should be difficult. In a few cases, students may master the more difficult knowledge first and then quickly master the simple knowledge based on it. The premise is that simple knowledge is not the only premise requirement for difficult knowledge. Students are generally assumed to learn one knowledge point at a time in learning trajectory research until they master all the knowledge points. Through the logical relationship between student attribute patterns in CDM, the possible learning trajectory of students can be drawn. The knowledge state of each participant is first obtained through parameter evaluation, which is the mastery of each attribute of the participant; then, the participants with the same knowledge state are classified and categorized to establish the trajectory relationship among the knowledge states (Wu et al., 2020). Figure 2 shows the learning trajectory map of 14 countries.

Figure 2 indicates the learning trajectories in 14 countries. The red solid line path is the main learning trajectory. It contains the largest proportion of participants with knowledge of all attribute patterns. The black dashed part is the secondary learning trajectory. Its proportion in the attribute patterns of the same level is slightly lower than the red part. However, when the ratio of the secondary to the main trajectory is excessively large (more than an order of magnitude), the existence of a secondary learning trajectory is not considered. Figure 2 indicates that Tunisia, Uruguay, Chile, and Romania have only one main learning trajectory. The other 10 countries have main and secondary learning trajectories. Nearly, all the national data support the frequency attribute as a prerequisite for other attributes. Taking Argentina as an example, students first master the first uncertainty attribute, followed by the fourth data handling attribute, then, the third statistical chart attribute, and finally, the second average attribute. However, in the student attribute patterns of Serbia, the ratio of (1,0,0,0) and (0,0,0,1) is nearly the same. It may indicate that Serbia has primary and secondary learning trajectories.

Figure 2 indicates that some countries have the same learning trajectories. For example, Bulgaria, Costa Rica, and Peru have the same learning trajectories. Mexico and Serbia have the same learning trajectories. Interestingly, from mastering non-attribute level to mastering two-attribute level, the main learning trajectories of Bulgaria, Costa Rica, and Peru are the secondary learning trajectories of Mexico and Serbia. The secondary learning trajectories of Bulgaria, Costa Rica, and Peru are the main learning trajectories of Mexico and Serbia. Mexico, Serbia, Colombia, and Jordan have the same learning trajectories from mastering non-attribute level to mastering two-attribute level, but different learning trajectories from mastering two-attribute level to mastering four-attribute level.

The first mastering attribute is uncertainty in 10 (e.g., the United Arab Emirates, Argentina, Chile, Colombia, Jordan, Kazakhstan, Mexico, Serbia, Tunisia, and Uruguay) out of 14 countries in mastering one-attribute level. Except for Kazakhstan and Romania, 12 countries all regard (1,0,0,1) as the main attribute in mastering two-attribute level, which means the mastering attributes are uncertainty and data handling. Except for Romania, Colombia, and Jordan, 11 countries all regard (1,0,1,1) as the main attribute in mastering three-attribute level, which means the mastering attributes are uncertainty, statistical chart, and data handling. None of the 14 countries regard the average attribute as a priority attribute. Only Romania regards statistical chart attribute as a priority attribute. This finding indicated that the best learning trajectory may be (0,0,0,0) → (1,0,0,0) → (1,0,0,1) → (1,0,1,1) → (1,1,1,1).

Research shows that some countries have only one primary learning trajectory, and some countries have a secondary learning trajectory. The learning trajectory of Romania is different from other countries. In another 13 different countries, a relatively uniform learning trajectory exists for the four statistical attributes. This result means that in statistical learning, the best learning order may be uncertainty → data handling → statistical chart → average. Additionally, teaching by this learning trajectory may be suitable for the learning of students. This learning trajectory is also the order of teaching in textbooks in some countries. For example, Chinese textbooks have adopted this order. The learning trajectories from the low to the top ends represent different ability levels, reflect the ability relationship among knowledge states, describe the development process of students, and show the clear development of trajectory and direction for students from low-level learning to high-level learning abilities (Wu et al., 2020). Therefore, the learning trajectory found in this research can be used not only to guide teaching but also to provide a basis for the remedial teaching of teachers.

## Discussion

Based on the data of PISA 2012 and CDM, this study calculated the attribute patterns of students in 14 countries in statistical knowledge and inferred the possible learning trajectories of students in statistical knowledge. The study found that the statistical knowledge learning trajectories of most of the 14 countries have certain common characteristics. The best learning order for the four attributes may be uncertainty → data handling → statistical chart → average. Nonetheless, the research also found special cases. The second attribute that Romania students master is average. This finding is different from all the other 13 countries. Additionally, the data of Romania do not show the existence of secondary learning trajectory. In the following research, we can consider comparing Romanian textbooks with other countries and perhaps determine an explanation for this phenomenon.

The average is the most difficult attribute in the four attributes. From a mathematical view, calculating the average is easy. However, from a statistical view, understanding how the average is used as a statistic to describe the population is difficult for students. Many pieces of research have revealed average as difficult for students (Rubin et al., 1991; Mokros and Russell, 1995; Bright and Friel, 1998; Shaughnessy, 2007; Kaplan et al., 2010). This research also proves this finding. Although statistics teaching in reality is more difficult and complex than imagined (Garfield and Ben-Zvi, 2007), teaching based on learning trajectory is one of the important methods to improve the effect of statistical learning (Garfield and Ben-Zvi, 2008). This research shows that the learning average after the other three attributes may be the most conducive to student learning.

The 2012 PISA study found that all participating countries have a considerable number of under-performing students, and significant differences exist in these under-performing students between different countries (OECD, 2016). This research shows that in 14 countries, Bulgaria, Kazakhstan, and the United Arab Emirates have relatively high proportions of students. Costa Rica and Peru had the highest proportion of students who did not master any attributes. However, Bulgaria, Costa Rica, and Peru have the same learning trajectory. It shows that even if the learning trajectory is the same, the level of mastery of the attributes of students in different countries may be different. Although studies have shown that IQ and the economy are positively correlated (Meisenberg and Woodley, 2013), the economy of Bulgaria is lower than that of Costa Rica and Peru.

Although this study uses CDM to conduct an in-depth analysis of the statistical items in PISA 2012, some aspects still require improvement. First, the exam items in different countries in the PISA are not exactly the same, and the open items are also limited. China, the United States, Japan, and other countries have fewer public statistical items in PISA 2012, and CDM cannot be used for estimation. Therefore, future research should consider obtaining information from other international examinations and exploring the learning trajectory in China, the United States, Japan, and other countries. Second, this research is a quantitative research based on big data. It requires comparison and confirmation using longitudinal research. Empirically, learning trajectories are stable over time; that is, the knowledge states that are more frequent in the past should also be the most frequent today. This question should be verified in future research. Third, the textbooks of different countries contain different statistical knowledge points. Future research needs to compare textbooks of different countries, summarize knowledge points, and then conduct statistical tests for more in-depth analysis.

## Data Availability Statement

The datasets presented in this study can be found in online repositories. The names of the repository/repositories and accession number(s) can be found in the article/Supplementary Material.

## Author Contributions

BJ designed the study. ZZ wrote this manuscript. HG provided technical support, and contributed to the manuscript writing and the continued revision provided by the reviewers. All authors contributed to the article and approved the submitted version.

## Conflict of Interest

The authors declare that the research was conducted in the absence of any commercial or financial relationships that could be construed as a potential conflict of interest.
